# Estrogen Modulation of B Cell Immunity: Implications for HIV Control and Therapeutic Strategies

**DOI:** 10.1002/cph4.70050

**Published:** 2025-09-12

**Authors:** John Kasibante, Todd T. Brown, Jordan E. Lake, Mohamed Abdel‐Mohsen

**Affiliations:** ^1^ Division of Infectious Diseases, Department of Medicine Feinberg School of Medicine, Northwestern University Chicago Illinois USA; ^2^ Center for Human Immunobiology, Department of Medicine Feinberg School of Medicine, Northwestern University Chicago Illinois USA; ^3^ Potocsnak Longevity Institute, Department of Medicine Feinberg School of Medicine, Northwestern University Chicago Illinois USA; ^4^ Johns Hopkins University Baltimore Maryland USA; ^5^ UTHealth Houston Houston Texas USA

**Keywords:** antibody, antibody‐producing cells, B cells, estrogen, glycosylation, HIV, sex hormones

## Abstract

Biological sex profoundly impacts HIV acquisition, disease progression, and persistence. Beyond genetic differences, sex hormones such as estrogen play multifaceted roles in shaping immune responses to HIV. However, the precise effects of estrogen and other sex hormones on the various components of the immune system, and their implications for HIV progression and persistence, remain poorly understood. Addressing these gaps is essential for developing strategies to improve the management of chronic HIV, especially in post‐menopausal cisgender women and transgender women. B cells are crucial for HIV control, primarily through the production of anti‐HIV antibodies. Emerging evidence suggests that estrogen exerts significant, yet underappreciated, effects on B cell function. However, the interactions between estrogen and B cells during HIV remain poorly characterized. This review explores current insights into the estrogen–B cell axis, emphasizing its role in modulating immune responses critical to HIV. Specifically, it examines estrogen's effects on B cell activation, antibody production, and antibody functionality, all of which can influence HIV control and disease progression. We also highlight key research gaps, including the impact of differential estrogen levels on immune‐mediated HIV control and the potential of estrogen modulators to enhance B cell‐driven immunity during HIV.

## Graphical abstract

Estrogen significantly modulates B‐cell immunity. We provide evidence for estrogen–B‐cell crosstalk (the estrogen–B‐cell axis) in regulating B‐cell function and shaping HIV acquisition, progression, and persistence, and highlight research gaps to guide hormone‐aware vaccines and therapies.

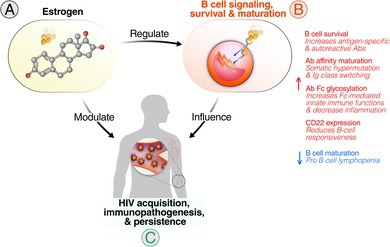

## 1 Introduction

Among the 39.9 million people living with HIV (PLWH) globally, cisgender women (CW) and girls constitute over half (53%) (UNAIDS 2023). Biological sex significantly influences HIV acquisition, progression, and persistence, partly due to inherent differences in immune function and response (Altfeld and Scully 2023; Gianella et al. 2016, 2022; Griesbeck et al. 2016; Scully 2018; Maskew et al. 2013; Patel et al. 2014). For example, CW face a higher risk of HIV acquisition through heterosexual intercourse compared to cisgender men (CM) (Scully 2018). In addition, in ovariectomized macaques, systemic estradiol or topical estriol thickens/cornifies the vaginal epithelium and markedly reduces SIV vaginal transmission, whereas susceptibility is higher in progesterone‐dominant states and with progestin analogs (Butler et al. 2015; Kersh et al. 2014; Smith et al. 2000). Disease progression also varies by sex, though findings are sometimes inconsistent. CW tend to experience lower viral load set points than CM; however, both groups exhibit equivalent rates of disease progression (Gandhi et al. 2002). Both CW and CM experience CD4^+^ T cell declines and inverted CD4^+^/CD8^+^ T cell ratios after HIV acquisition. However, CW often show faster and more sustained CD4^+^ T cell recovery after initiating antiretroviral therapy (ART) than CM (Maskew et al. 2013). Conversely, some studies report higher levels of immune activation and inflammation markers in CW compared to CM, even during virologic suppression with ART (Santinelli et al. 2020; Mathad et al. 2016). These heightened inflammatory responses may contribute to the development of aging‐ and inflammation‐associated comorbidities, such as cardiovascular disease (Hunt et al. 2016; Raghavan et al. 2017; Triant et al. 2007; Fitch et al. 2013; Chow et al. 2018). Sex differences also extend to HIV persistence. For instance, CW may exhibit lower levels of cell‐associated HIV DNA and RNA, residual viremia, and replication‐competent virus than CM; however, these findings are not consistent in the literature (Cuzin et al. 2015; Gandhi et al. 2017; Prodger et al. 2020; Scully et al. 2019). Additionally, in CM, HIV reservoirs decline with age, whereas in CW, reservoirs initially decrease until menopause, after which they expand (Gianella et al. 2022).

The drivers of these sex‐related differences in immunological responses in PLWH are likely multifactorial, encompassing behavioral, genetic, epigenetic, and hormonal factors (Dillon et al. 2011; Scofield et al. 2008; Hewagama et al. 2013; Sawalha et al. 2012; Khan and Ahmed 2015; Wira et al. 2015). Notably, recent studies highlight the pivotal role of sex hormones, particularly estrogen, in mediating many of these differences (Butler et al. 2015; Kersh et al. 2014; Smith et al. 2000; Allred et al. 2006; Bick and Hapgood 2018; Das et al. 2018; Heron et al. 2009; Kettelhut et al. 2022; Lee et al. 2004; Martinez et al. 2023; Peters et al. 2024; Szotek et al. 2013; Wilson et al. 2008). Estrogen not only directly inhibits HIV replication and transcriptional activity (Szotek et al. 2013; Henderson et al. 2012; Narasipura et al. 2012), but also can exert significant indirect effects on HIV by modulating several immunological functions critical for HIV/SIV control (Das et al. 2011; Goode et al. 2014; Hahn et al. 2025; Lu et al. 1999, 2002). Despite these insights, the specific effects of estrogen on distinct immune system components and their implications for HIV‐associated immune responses remain poorly understood. Addressing this knowledge gap is essential for advancing therapeutic interventions aimed at mitigating aging‐associated comorbidities and HIV persistence. Many experimental therapies targeting these conditions focus on modulating the host immune system. Therefore, developing inclusive and effective treatments relies on a comprehensive understanding of sex‐ and gender‐specific immune mechanisms.

**FIGURE 1:**
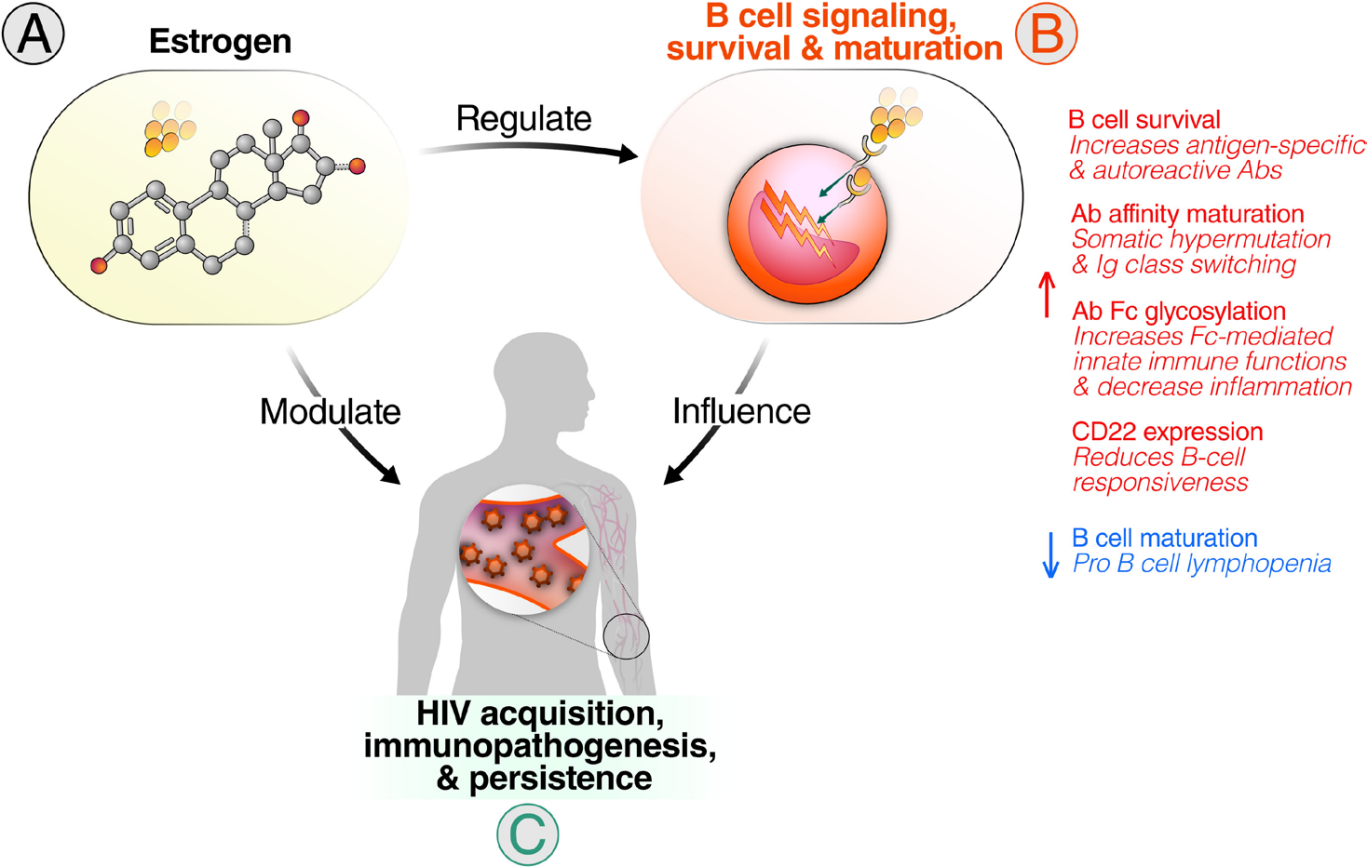
The estrogen–B cell axis in HIV. A dynamic interplay exists between estrogen and B cell function in the context of HIV. (A) Estrogen regulates multiple facets of (B) B cell biology, including signaling, survival, differentiation, and antibody production. Estrogen receptors (ERα and ERβ) mediate these effects through genomic and non‐genomic pathways, influencing transcriptional activity and intracellular signaling cascades. Estrogen enhances antibody function by promoting class switching, somatic hypermutations, and Fc glycosylation, which in turn modulates antibody‐dependent effector functions such as ADCC, ADCP, and ADCD. (C) These processes are crucial for antibody‐mediated immune responses against HIV. The modulation of B cell responses by estrogen could have significant consequences for HIV acquisition, immune activation, and viral reservoir dynamics. Together, these interactions highlight the estrogen–B cell axis as a key immunoregulatory pathway, with potential important implications for HIV vaccine responses, immunopathogenesis, and persistence.

This review focuses on the influence of estrogen on B cell function during HIV. B cells are central to HIV control through their production of HIV‐specific antibodies as well as other functions (de Bree and Lynch 2016; Doria‐Rose and Connors 2009; Moir and Fauci 2009; Siewe and Landay 2012). Emerging evidence indicates that estrogen significantly modulates B cell functions, including activation, differentiation, and antibody production (Medina et al. 2001; Grimaldi et al. 2002; Getahun et al. 2016; Clark and Giltiay 2018; Pauklin et al. 2009; Gupta et al. 2023; Shao et al. 2017; Giron et al. 2024). By integrating the understanding that estrogen modulates B cell function with the established role of B cells in influencing HIV disease progression, it is plausible that the interaction between estrogen and B cells (the estrogen–B cell axis) plays a pivotal role in shaping immune responses to HIV (Figure 1). In this review, we first examine how estrogen influences B cell biology, highlighting the mechanisms underlying the estrogen–B cell axis. Next, we discuss the role of B cell responses in HIV acquisition, progression, and persistence. We then explore how the estrogen–B cell axis could contribute to modulating immune responses to HIV. Finally, we identify key research gaps, including the impact of differential estrogen levels across age and genders on B cell‐mediated immune responses in PLWH. These insights may guide the development of therapeutic strategies tailored to optimize immune responses across sexes and genders in the context of HIV.

## 2 The Interaction Between Estrogen and B Cells (the Estrogen–B Cell Axis)

B cells, particularly plasmablasts and plasma cells, key producers of antibodies, express estrogen receptors (ERs). Estrogen can influence B cell signaling and function through both genomic and non‐genomic pathways. Estrogen can cross cell and nuclear membranes to bind intracellular ERs (ERα and ERβ) or interact with G‐protein‐coupled receptors on the plasma membrane. Estrogen binding to ERα or ERβ recruits cofactors such as AF‐2, enabling dimerization of estrogen‐ER complexes, which then bind to estrogen response elements (EREs) in the promoter regions of target genes. This signaling pathway mediates the classical estrogen‐ER‐ERE actions (Kushner et al. 2000). In ERE‐independent actions, estrogen‐ER dimers interact with transcription factors via protein–protein interactions without directly binding EREs. For instance, genes like IGF‐1 and Cyclin D1 are upregulated through interactions with transcription factors such as AP‐1, involving Jun and Fos family proteins (Kushner et al. 2000; Zwijsen et al. 1997; Bjornstrom and Sjoberg 2005). Estrogen also mediates ligand‐independent actions by directly activating signal transduction pathways, including protein kinase activation, which subsequently modulate transcription factor activity (Bjornstrom and Sjoberg 2005). The fourth mechanism by which estrogen mediates its functions is through the rapid non‐genomic actions, such as activation of endothelial nitric oxide synthase (eNOS) (Chen et al. 1999). These rapid estrogen responses are thought to be mediated via G‐protein‐coupled ERs (Ahmadian Elmi et al. 2023).

Through these signaling pathways, estrogen influences multiple stages of B cell development and activation. For instance, during early B cell development, estrogen decreases lymphopoiesis by impairing the progression of pro‐B cells to pre‐B cells in the bone marrow (Medina et al. 2001). However, in the later splenic B cell stages, estrogen promotes the survival of transitional and mature B cells by upregulating the Bcl‐2 gene, which in turn stabilizes the mitochondrial membranes and prevents mature B cells from undergoing intrinsic apoptosis through the inhibition of cytochrome C release. This protective mechanism extends to both normal and autoreactive B cells and thus reduces B cell negative selection. Pre‐clinical models in mice show that sustained physiological levels of estrogen are crucial for the rescue and activation of autoreactive B cells (Bebo Jr. et al. 2001). Estrogen also regulates B cell activation by modulating SHP‐1 and CD22 expression, both of which negatively regulate B cell receptor signaling (Grimaldi et al. 2002; Getahun et al. 2016; Clark and Giltiay 2018). By fine‐tuning the expression of these molecules, estrogen alters the activation threshold of B cells and thus directly impacts their immune responses.

Estrogen also increases antibody secretion and promotes antibody affinity maturation. Estrogen upregulates activation‐induced cytidine deaminase (AID), a key enzyme essential for somatic hypermutation and class switch recombination. During the primary immune response, AID facilitates random mutations in the immunoglobulin variable regions, thereby enhancing the antigen‐binding affinity of antibodies. Estrogen‐ER complexes bind directly to the AID promoter, increasing mRNA expression and subsequently elevating AID protein levels (Pauklin et al. 2009). Short‐term estrogen exposure has been shown to upregulate AID mRNA in murine models (Pauklin et al. 2009). Consistently, long‐term estrogen deprivation, as observed in ovariectomized mice, results in reduced levels of class switched immunoglobulins, IgG and IgA, and indicates a pivotal role estrogen plays in antibody affinity maturation (Gupta et al. 2023; Shao et al. 2017).

Beyond influencing antibody diversity, estrogen also impacts antibody functionality, particularly Fc‐mediated innate immune functions. These include antibody‐dependent cellular cytotoxicity (ADCC), phagocytosis (ADCP), complement deposition (ADCD), and the modulation of inflammatory responses. The functionality of these processes is significantly shaped by the glycosylation profiles of antibody Fc regions, which mediate interactions between the Fc region and its corresponding Fc receptors (Goede et al. 2014; Junttila et al. 2010; Scott et al. 2012; Sondermann and Szymkowski 2016). For example, the anti‐inflammatory activity of intravenous immunoglobulins (IVIGs) is heavily dependent on the sialic acid content of their Fc regions (Goede et al. 2014; Junttila et al. 2010; Scott et al. 2012; Sondermann and Szymkowski 2016; Ahmed et al. 2014; Anthony, Nimmerjahn, et al. 2008; Anthony, Wermeling, et al. 2008; Kaneko et al. 2006; Washburn et al. 2015; Anthony and Ravetch 2010; Nimmerjahn and Ravetch 2007; Chan and Carter 2010; Karsten et al. 2012; Masuda et al. 2007). Estrogen treatment in murine models has been shown to enhance the levels of anti‐inflammatory glycans, such as galactose and sialic acid, on IgG Fc glycans (Gupta et al. 2023), in post‐menopausal murine models. Consistently, post‐menopausal women exhibit a loss of galactosylated glycans on IgG (Gupta et al. 2023; Giron et al. 2024), and humans treated with estrogen show alterations in IgG glycomic profiles (Ercan et al. 2017; Juric et al. 2020; Mijakovac et al. 2021), which can affect antibody functions beyond neutralization. These findings highlight the potential role of estrogen in mediating the structural and functional properties of antibodies.

Collectively, these findings underscore the multifaceted influence of estrogen on B cells, spanning development, activation, antibody maturation, and glycosylation, and highlighting its critical role in shaping humoral immune responses.

## 3 Role of B Cells in HIV Control

B cells play an essential role in controlling HIV across its various stages, primarily through antibody production, co‐stimulatory signaling, and the secretion of key cytokines. For instance, one major focus on preventing HIV acquisition has been on harnessing B cells for the development of an effective HIV vaccine. Such a vaccine relies on the ability of B cells to produce broadly neutralizing antibodies (bNAbs) upon antigenic stimulation. Given the extensive genetic diversity of HIV, multiple bNAbs are often required to neutralize virions and prevent HIV acquisition (Sneller et al. 2022; Corey et al. 2021). The failure of many vaccine candidates to prevent HIV infection in clinical settings has been largely attributed to their inability to elicit these bNAbs, which are also rarely elicited during living with HIV (N'Guessan et al. 2024; Gray et al. 2024). Beyond direct neutralization, there is evidence that Fc‐mediated effector functions, such as ADCC, also contribute to vaccine efficacy. For example, in the RV144 Thai trial, ADCC responses correlated with modest protective efficacy (Haynes et al. 2012). Consequently, both the quantity and quality of B‐cell‐derived antibodies are crucial for the success of future HIV vaccine strategies.

During early HIV, plasmablasts and plasma cells are activated and begin to secrete antibodies against the virus (Richman et al. 2003). While anti‐HIV antibodies can be detected in serum as early as 4 days after p24 antigen becomes measurable and after viremia has begun to decline from its peak, neutralizing antibodies usually appear much later, at around 12 weeks post‐transmission (Richman et al. 2003; Robb et al. 2016; Moore et al. 1994; McMichael et al. 2010; Frost et al. 2005). It thus remains unclear whether the early decline in virus levels is driven in part by low‐level, early antibody responses or by other factors, highlighting the unresolved role of B‐cell‐mediated control in the earliest stages of HIV.

During chronic HIV, significant dysregulation in B cell homeostasis and functionality becomes evident. For instance, chronic HIV is characterized by B cell lymphopenia alongside expansions of CD19^+^CD21^+/−^CD10^+^ immature/transition B cells and immune‐exhausted CD19^+^CD10^−^CD21^−/lo^ tissue‐like memory (TLM) B cells (Moir et al. 2008). The CD19^+^CD21^+/−^CD10^+^ transitional subset exhibits low levels of Bcl‐2 and is highly prone to apoptosis (Moir and Fauci 2013). Furthermore, these CD10^+^ cells are largely unresponsive to various stimuli, including HIV virions, undermining their potential to contribute to viral control (Malaspina et al. 2006). Chronic HIV also diminishes the population of CD19^+^CD10^−^CD21^+^CD27^+^ resting memory (RM) B cells (Moir et al. 2001, 2008), a subset that plays an essential role in sustaining humoral immunity against both HIV and other pathogens. The depletion of RM B cells, coupled with ongoing viral evolution, impairs the affinity maturation required to generate potent neutralizing antibodies (Richman et al. 2003; Frost et al. 2005).

Despite this dysfunction, multiple lines of evidence indicate that B cell function, and the quality and quantity of HIV‐specific antibodies they produce, can still influence viral control during chronic HIV, under ART, and following treatment interruption. For example, autologous neutralizing antibody responses against replication‐competent virus during ART have been shown to correlate with viral control after discontinuation of ART (Van Gulck et al. 2012; Blazkova et al. 2021; Bertagnolli et al. 2020; Garcia et al. 2024). Furthermore, some post‐treatment controllers, individuals who maintain viral remission for extended periods after stopping ART (Giron et al. 2025), exhibit bNAbs that may contribute to their ability to contain the virus (Molinos‐Albert et al. 2023). Beyond neutralization capacity, the glycomic profiles of HIV‐specific antibodies, which critically modulate their Fc‐mediated effector functions (Goede et al. 2014; Junttila et al. 2010; Scott et al. 2012; Sondermann and Szymkowski 2016; Baum et al. 1996; Bruel et al. 2016; Chung et al. 2011; Lee and Kent 2018), have been linked to the timing and likelihood of viral rebound after treatment interruption (Giron et al. 2020, 2021; Offersen et al. 2020). Collectively, these findings underscore the importance of optimizing B cell responses for improved HIV control during chronic infection, both while on ART and upon ART cessation.

Although best known for antibody production, B cells also serve as professional antigen‐presenting cells (APCs) that activate T cells. In PLWH, especially those with uncontrolled viremia, chronic stimulation leads to an accumulation of immune‐exhausted B cells characterized by downregulation of co‐stimulatory molecules (CD80, CD86) and upregulation of inhibitory receptors, thus undermining B–T cell crosstalk (Moir and Fauci 2013; Malaspina et al. 2003; Jiang et al. 2008). Reduced CD80 and CD86 impair the binding of CD28 on T cells, an interaction critical for antigen‐specific responses. Accumulated TLM B cells in PLWH with viremia often overexpress Fc receptor–like 4 (FCRL4), which interferes with immune synapse formation and B‐cell receptor signaling via recruitment of phosphatases (SHP‐1, SHP‐2) and inhibitory receptors like CD22 and PDL‐1 (Sohn et al. 2011; Ehrhardt et al. 2003; Rinaldi et al. 2017). Taken together, these disruptions in co‐stimulatory signaling can exacerbate systemic immune dysregulation and diminish the overall effectiveness of immune responses in PLWH.

B cells can also fuel chronic inflammation through the production of pro‐inflammatory cytokines such as IL‐6 and TNF‐α (Moir and Fauci 2013). Moreover, aberrant glycosylation of bulk antibodies can amplify inflammation by inhibiting the normal anti‐inflammatory pathways mediated through REST‐dependent suppression of NF‐κB signaling (Chakraborty et al. 2025). PLWH, even with long‐term ART, exhibit aberrant bulk antibody glycosylation with reduced anti‐inflammatory glycans, potentially contributing to persistent inflammation and limited control of virally infected cells (Giron et al. 2024). Dysfunctional B cells additionally heighten the risk of autoimmunity through accumulation of T‐bet^+^ CD11c^+^ age‐associated B cells (ABCs) (Mouat and Horwitz 2022; Ruggiero et al. 2022; Mouat et al. 2022). ABCs develop early during HIV and continue to accumulate during chronic HIV and are crucial mediators in viral infections related to autoimmunity and age‐related diseases like atherosclerosis (Mouat and Horwitz 2022; Comarmond et al. 2019; Pattarabanjird et al. 2023; Ait‐Oufella et al. 2010).

Finally, B cells are crucial at mucosal sites, particularly in gut‐associated lymphoid tissue, for producing immunoglobulin A (IgA), thereby maintaining barrier integrity and preventing microbial translocation (Mestecky et al. 2004; Lindner et al. 2015; Planchais et al. 2023; Schafer et al. 2002). Excessive microbial translocation drives inflammation, contributes to inflammation‐associated comorbidities in PLWH, and is exacerbated by HIV‐driven depletion or dysfunction of mucosal B cells (Mestecky et al. 2004; Planchais et al. 2023). Thus, preserving or restoring mucosal B cell compartments and, in turn, IgA responses, represents an important strategy for alleviating chronic immune activation in PLWH.

Together, these data highlight the significant, multifaceted impacts of B cells in modulating the overall efficiency of the immune system against HIV and other opportunistic diseases, which is critical for disease progression of PLWH. Additionally, B cells play key roles in modulating inflammation and autoimmunity, which are critical for the health span of PLWH.

## 4 Role of the Estrogen‐B Cell Axis in Modulating Immune Responses During HIV

Building on the established role of B cells in HIV disease progression (Section 3) and the influence of estrogen on B cell function (Section 2), it is plausible that the estrogen–B cell axis plays a critical role in shaping immune responses to HIV (Figure 1). For instance, this axis may influence both the quantitative and qualitative aspects of anti‐HIV antibodies, ultimately affecting viral control and immune regulation in PLWH. Indeed, in female rhesus macaques, ovarian steroids modulate humoral immunity: in vivo, the frequency of antibody‐secreting cells (ASCs) in genital and systemic lymphoid tissues peaks peri‐ovulation; in vitro, using rhesus PBMC cultures, estradiol increases, whereas progesterone decreases, ASC frequency in a CD8^+^ T‐cell–dependent manner (Lu et al. 2002).

One of the major challenges in HIV prevention and cure is the generation of anti‐HIV antibodies with broad neutralization that are capable of effectively controlling the virus. Anti‐HIV vaccination strategies and natural HIV acquisition rarely elicit sufficient levels of these antibodies, as their development requires extensive somatic hypermutation, a process driven by AID in germinal centers (Wiehe et al. 2018). Additionally, generating such polyreactive antibodies necessitates an immune environment permissive to the survival of polyreactive B cells (Haynes et al. 2005, 2023). Given that estrogen enhances AID expression and reduces B cell tolerance, promoting the survival of polyreactive B cells (Pauklin et al. 2009), it is conceivable that estrogen may also support the production and persistence of B cells that can produce such anti‐HIV antibodies. This suggests that estrogen may be a previously underappreciated factor influencing the development and maintenance of potent anti‐HIV antibody responses. Consistent with this concept, female rhesus macaques exhibit hormone‐linked changes in B‐cell secretory activity and antibody‐secreting cell frequencies across the cycle (Lu et al. 2002). However, the role of estrogen in modulating B cell responses during HIV vaccination, exposure, or infection, and the impact of these effects on the quantity and quality of anti‐HIV antibodies across different reproductive stages and genders, remains unknown. Further research is needed to clarify how estrogen levels shape B cell‐mediated immunity in these contexts.

Beyond influencing the neutralizing capacity of HIV‐specific antibodies, the estrogen–B cell axis also has the potential to modulate their functional properties. As discussed earlier, estrogen significantly affects antibody glycosylation (Gupta et al. 2023), altering the Fc glycan profile in ways that can enhance Fc‐mediated effector functions such as ADCC, ADCP, and ADCD. These effector functions are critical for immune clearance of cells with HIV and could be influenced by fluctuations in estrogen levels. Additionally, estrogen‐driven changes in antibody glycosylation may modulate inflammatory properties, potentially affecting chronic immune activation in PLWH. However, the extent to which differential estrogen levels across ages and genders influence the qualitative features of HIV‐specific and bulk antibodies, and how these changes impact HIV control and immunopathogenesis, remain an open question.

Beyond HIV‐specific antibodies, estrogen can also influence broader humoral immune responses against other antigens, with significant implications for the overall health of PLWH. Notably, vaccine responses are known to be influenced by estrogen levels (Hahn et al. 2025), with differences in vaccine efficacy between sexes diminishing with age (Dhakal et al. 2024). In murine models, estrogen supplementation in post‐menopausal and aged mice has been shown to enhance vaccine responses (Dhakal et al. 2024). In rhesus macaques, exogenous estrogen enhances T cell activation after mRNA vaccination (Hahn et al. 2025). Given that PLWH often exhibit suboptimal vaccine responses (Hoft et al. 2024; El Chaer and El Sahly 2019; Crum‐Cianflone et al. 2011), harnessing the estrogen–B cell axis could offer new strategies for improving vaccine‐induced immunity and/or responses to other infections in this population.

Finally, estrogen plays a role in shaping mucosal immunity, particularly through its effects on IgA production. Studies in mice have demonstrated that estrogen increases the frequency of IgA‐secreting B cells by directly promoting class switching to IgA (Lagerquist et al. 2008). Parallel evidence in rhesus macaques shows that cervicovaginal IgG and IgA levels fluctuate with the menstrual cycle (highest during menses and lowest peri‐ovulation) supporting hormonal control of genital‐tract humoral defenses in primates (Lu et al. 1999). Moreover, peri‐ovulatory tissues show higher frequencies of ASCs, linking cyclical steroid levels to local B‐cell function (Lu et al. 2002). Although this mechanism remains to be fully explored in humans, it holds potential implications for restoring mucosal IgA levels in PLWH. Given the role of IgA in maintaining gut barrier integrity and preventing microbial translocation, a key driver of chronic inflammation in HIV, estrogen's effects on IgA production could have significant consequences for systemic immune activation and comorbidities in PLWH.

In summary, the estrogen–B cell axis represents a potentially important but understudied mechanism influencing immune responses in PLWH. While current evidence suggests that estrogen can modulate antibody production, glycosylation, and effector functions, its impact on B cell‐mediated immunity in PLWH across different ages and genders remains unclear. Further investigation is warranted to determine whether leveraging the estrogen–B cell axis could improve antibody‐mediated immunity, vaccine responses, and inflammation regulation in PLWH. Understanding these interactions could open new avenues for therapeutic interventions tailored to optimize immune responses across sexes and genders in the context of HIV.

## 5 Potential Therapeutic Applications of the B Cell–E2 Axis in HIV

Estrogen exerts both protective and potentially deleterious effects on B cells, enhancing beneficial immune responses while also promoting the survival of autoreactive B cells (Kanda and Tamaki 1999; Sthoeger et al. 1988; Cohen‐Solal et al. 2008; Malkiel et al. 2018; Suurmond et al. 2016). Consequently, therapeutic strategies that target the estrogen‐B cell axis must carefully balance leveraging estrogen's immunomodulatory properties against its risks. Beyond alleviating menopausal symptoms in CW with HIV, estrogen demonstrates notable immunoregulatory effects that could be harnessed to modulate HIV. For example, estrogen has been shown to inhibit HIV replication and reduce the susceptibility of CD4^+^ T cells and macrophages to HIV (Szotek et al. 2013). Additionally, higher endogenous estrogen levels in CW have been linked to smaller inducible HIV RNA reservoirs compared to CM (Das et al. 2018), suggesting that estrogen may help restrict HIV persistence and reservoir size.

Selective ERβ agonists, such as S‐Equol, offer another promising avenue for clinical intervention. S‐Equol has been shown to mitigate HIV‐associated neurocognitive impairment in transgenic mouse and rat models (Bertrand et al. 2015; McLaurin et al. 2020). Notably, S‐Equol is an enantiomer of Equol, a compound generated by the gut microbiota from soy‐derived phytoestrogens. Its neuroprotective effects have also been observed in some people with Alzheimer's disease (Wilkins et al. 2017). Since gut microbiota are partly regulated by IgA‐secreting B cells, these findings highlight a potential interconnected role of the estrogen‐B cell axis in modulating gut health, systemic immunity, and neuroinflammatory pathways in PLWH.

Despite these potential benefits, estrogen‐based therapies carry recognized adverse effects, especially in CM and transgender women (TW). In one case–control study, TW receiving gender‐affirming estrogen therapy exhibited a higher prevalence of cardiovascular disease, myocardial infarction, and type 2 diabetes compared to controls (Wierckx et al. 2013). Moreover, while PLWH generally have lower rates of certain autoimmune disorders, estrogen's propensity to promote the survival of autoreactive B cells could elevate the risk of autoimmunity among these individuals (Virot et al. 2017).

This dual nature of estrogen underscores the need for a deeper understanding of the precise mechanisms through which estrogen influences B cell biology and, by extension, HIV pathogenesis. Advances in this area could facilitate the development of targeted interventions, such as selective estrogen receptor modulators (SERMs) or tissue‐specific delivery methods, that maximize the immunotherapeutic potential of estrogen while minimizing harmful outcomes. Ultimately, refining our knowledge of the estrogen‐B cell axis may pave the way for novel, sex‐ and gender‐specific strategies to improve immune function and curb HIV persistence in PLWH.

## 6 Conclusion

Estrogen exerts wide‐ranging effects on immunity and other organ systems, including the central nervous, cardiovascular, and skeletal systems. This review focused on the estrogen–B cell axis to illustrate how hormone‐mediated modulation of a single immune cell population can profoundly impact HIV acquisition, pathogenesis, and control. However, a more holistic understanding of estrogen's influence, encompassing diverse immune cell types, organ systems, and life stages across sexes and genders, is critically needed to fully harness its beneficial effects while minimizing potential risks. Currently, our understanding of how sex hormones shape immune responses, both within and beyond HIV, remains incomplete.

While estrogen can enhance B cell responses by enhancing antibody production, affinity maturation, and Fc‐mediated effector functions, it may also exacerbate immune dysregulation, particularly in individuals predisposed to autoreactivity or other comorbidities. These dual, often conflicting effects highlight the complexity of estrogen's immunological influence. Evidence to date highlights an underappreciated role for estrogen in B cell biology that can affect multiple layers of HIV pathophysiology, from initial viral acquisition to chronic inflammation and viral persistence under ART or following treatment interruption. Moving forward, research should clarify the precise mechanisms by which estrogen modulates immunity, as well as determine whether selective estrogen receptor modulation can mitigate inflammation, limit HIV reservoirs, and promote beneficial immune responses in PLWH.

Therapeutic interventions that harness the positive aspects of estrogen, such as improved vaccine responses and enhanced production of protective antibodies, must also guard against potential risks like heightened autoimmunity or cardiovascular complications. This balance is especially pertinent for groups receiving gender‐affirming hormone therapy, post‐menopausal women undergoing hormone replacement therapy, older PLWH, and others who might benefit from SERMs. Ultimately, a holistic approach that integrates the broader physiological roles of estrogen with the specific context of HIV will be vital for designing novel, inclusive, and effective immunotherapeutic strategies.

## Author Contributions

J.K. and M.A.‐M. wrote the review. J.E.L. and T.T.B. provided consultancy and manuscript revisions.

## Ethics Statement

The authors have nothing to report.

## Consent

The authors have nothing to report.

## Conflicts of Interest

The authors declare no conflicts of interest.

## Data Availability

The authors have nothing to report.
